# The Role of Lithium in Management of Endocrine Tumors—A Comprehensive Review

**DOI:** 10.3389/fonc.2019.01092

**Published:** 2019-10-18

**Authors:** Shilpa Thakur, Andrew Tobey, Joanna Klubo-Gwiezdzinska

**Affiliations:** Metabolic Disease Branch, National Institute of Diabetes and Digestive and Kidney Diseases, National Institutes of Health, Bethesda, MD, United States

**Keywords:** lithium, endocrine tumors, mechanism of action, pre-clinical evidence, clinical evidence

## Abstract

**Background:** Epidemiological data reveal that treatment with lithium, a mood stabilizer, is associated with decreased incidence and mortality of certain cancer types, such as melanoma. Therefore, repositioning of lithium as an anticancer agent has emerged as a promising strategy in oncology. Since lithium affects the physiology of several endocrine tissues, the goal of this study was to analyze the role of lithium in the pathogenesis and treatment of tumors of the endocrine system.

**Methods:** The databases of PubMed, EMBASE, MEDLINE, were searched from January 1970 through February 2019 for articles including the keywords “lithium and”—“thyroid cancer,” “thyroid nodule,” “parathyroid adenoma,” “parathyroid carcinoma,” “pituitary adenoma,” “pituitary neuroendocrine tumor,” “neuroendocrine tumor,” “carcinoid,” “adrenal adenoma,” “adrenal carcinoma,” “pheochromocytoma/paraganglioma.” Preclinical *in vitro* and *in vivo* studies as well as case series, retrospective cohort studies and prospective trials were selected for the analysis.

**Results:** Treatment with lithium has been associated with a higher prevalence of thyroid enlargement, hypothyroidism and increased calcium levels due to parathyroid adenoma or hyperplasia, as one of the mechanisms of its action is to stimulate proliferation of normal follicular thyroid and parathyroid cells via activation of the Wnt signaling pathway. Supratherapeutic concentrations of lithium decrease the activity of glycogen synthase kinase-3β (GSK-3β), leading to cell cycle arrest in several *in vitro* cancer models including medullary thyroid cancer (TC), pheochromocytoma/paraganglioma and carcinoid. Growth inhibitory effects of lithium *in vivo* have been documented in medullary TC xenograft mouse models. Clinically, lithium has been used as an adjuvant agent to therapy with radioactive iodine (RAI), as it increases the residence time of RAI in TC.

**Conclusion:** Patients chronically treated with lithium need to be screened for hypothyroidism, goiter, and hyperparathyroidism, as the prevalence of these endocrine abnormalities is higher in lithium-treated patients than in the general population. The growth inhibitory effects of lithium in medullary TC, pheochromocytoma/paraganglioma and carcinoid were achieved with supratherapeutic concentrations of lithium thus limiting its translational perspective. Currently available clinical data on the efficacy of lithium in the therapy of endocrine tumors in human is limited and associated with conflicting results.

## Introduction

Lithium (lithos for stone in Greek) was discovered in the nineteenth century and it's salt—lithium carbonate has been widely used to treat bipolar disorder (1). Interestingly, patients chronically exposed to lithium carbonate were also characterized by a lower incidence of Alzheimer disease (2). These clinical observations prompted an investigation of the pleiotropic effects of lithium carbonate in human physiology and pathology. One of the important findings was that lithium affects glycogen synthase kinase-3β (GSK-3β) signaling pathway. GSK-3β functions as an important regulatory kinase and its hyperactivity have been linked to several disorders such as Alzheimer disease, schizophrenia (3), diabetes (4), and cancer (5, 6). Therefore, the reposition of lithium as an inhibitor of GSK-3β has emerged as a potential tool in cancer treatment. This interest is further supported by functional studies documenting the effect of lithium on numerous other pathways, including the WNT/β-catenin pathway (7), activation of CREB (cAMP-response-element-binding protein), interaction with TORC1 (1, 8) immunomodulation of lymphokine-activated killer cells and its effects on cell metabolism (glycolysis) (9–11). All of these cellular processes play an important role in pathogenesis of tumors of the endocrine system (12–20). Most of the studies analyzing the effects of lithium in the management of cancers have been primarily focused on thyroid cancer. Besides endocrine tumors, there are few reports where lithium use was associated with growth inhibition of melanoma (9, 21), prostate (22), and hepatocellular carcinoma cells (23, 24). In a retrospective cohort study, lithium exposure was associated with reduced melanoma risk and mortality (25). In contrary to this, lithium exposure increased the incidence of renal tumors in the lithium-treated patients (26).

The therapy of a subset of endocrine cancers is very challenging and the goal of this review is to provide an overview of the potential utility of lithium in the management of endocrine tumors based on published pre-clinical and clinical data.

## Lithium and Benign and Malignant Thyroid Nodules

### Epidemiology

As the use of lithium for the manic state of the bipolar disorder increased, so did the appearance of goiters and hypothyroidism, suggesting a role of lithium in the synthesis and storage of thyroid hormones (27). The prevalence of goiter has been reported to be as common as 4–60% (28–33) and hypothyroidism was observed in up to 6–52% of patients taking lithium (28, 34–46) while its prevalence in general population varies between 4.8–28 and 4.6–9.5%, respectively (47–49). On the other hand, lithium can also cause damage to the thyroid cell with consequent signs and symptoms of thyroiditis (28, 50). Interestingly, lithium is concentrated by the thyroid at levels 3–4 times higher than in the circulation (51). Lithium was found to increase the intrathyroidal iodine content as well as to inhibit release of thyroid hormones from the thyroid into the circulation due to altered tubulin polymerization in thyrocytes (28, 52, 53). In fact, the latter property has been used to treat patients with hyperthyroidism. Similar mechanisms have been proposed to be utilized to extend the retention of radioactive iodine (RAI) within the thyroid gland to treat either benign toxic multinodular goiters, Graves' disease or differentiated thyroid cancer (54–60). The application of lithium as adjuvant therapy for metastatic thyroid cancer is particularly interesting in view of the fact that the incidence of differentiated thyroid cancer (DTC) is rising more rapidly than any other type of cancer and that RAI-non responsive metastatic DTC is characterized by a poor outcome (61, 62).

### Mechanism of Lithium Action—Pre-clinical Evidence

**Figure 1:**
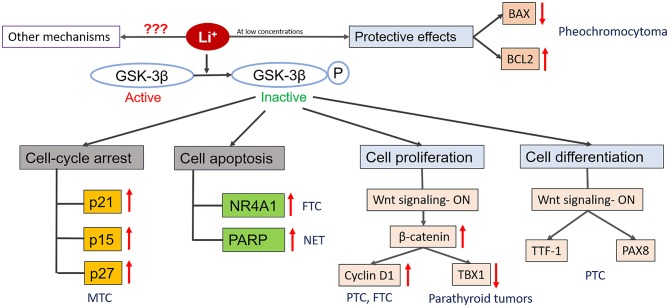
Diagrammatic representation of known mechanisms of action of lithium in endocrine cancers. Lithium treatment inactivates GSK-3β by phosphorylating it. Inactivation of GSK-3β can promote cell death by increasing the expression of cyclin-dependent kinase inhibitors (p21, p15, p27) that promotes cell cycle arrest and/or by increasing the expression of transcription factors (NR4A1) which induce expression of pro-apoptotic genes. Depending upon the cell types and concentration, lithium can also promote cellular proliferation by activating Wnt/β-catenin signaling pathway and its downstream mediators which have the ability to regulate the expression of various genes that play important role in cell growth and survival (e.g., Cyclin D1, TBX1). Lithium treatment can also promoter cell differentiation by increasing the expression of tissue specific transcription factors (TTF-1, PAX8). Also, lithium treatment at low concentration provide protective effects to the cells against toxic compounds by inhibiting the expression of BAX (pro-apoptotic gene) and promoting the expression of BCL2 (anti-apoptotic gene). Besides these, there might be other mechanisms of action involved which are currently unknown.

Since therapy with lithium has been associated with a high prevalence of goiter, there have been several studies evaluating the growth stimulatory effects of lithium on normal follicular cells. *In vivo* studies in rat models indicated that exposure to lithium resulted in an increase in follicular diameter and a decrease in follicle cell height (63). Functional *in vitro* models revealed that Wnt/β-catenin signaling may be important in lithium-associated goiter, as lithium significantly increased human thyrocyte proliferation mediated by Wnt/β-catenin pathway (64–66). Wnt/ β-catenin pathway plays a crucial role in organ formation during embryonic development, stem cell renewal and cell survival (67, 68). Aberrant activation of this pathway has been seen in many cancers. This pathway can regulate multiple target genes which play important roles in various physiological processes like proliferation, transformation and differentiation (67, 68). Gilbert-Sirieix et al. documented that the Wnt/β-catenin pathway regulates expression of one of the most important transcription factors in the thyroid gland, thyroid-transcription factor 1 (TTF1), in the papillary thyroid cancer (PTC) cell line TPC1 and in patient-derived PTC tumors (20). The activation of Wnt signaling by lithium chloride at the concentration of 5 and 20 mM induced TTF-1 gene and protein expression, suggesting a role of lithium in tumor differentiation (20). This observation is particularly important in view of the fact that de-differentiation is often observed in thyroid cancer, during which tumors progressively lose the expression of thyroid-specific genes such as sodium-iodine symporter (NIS), paired-box gene 8 (PAX8) or thyroglobulin (Tg) (69). Dupain et al. observed that treatment with 20 mM lithium resulted in upregulation of thyroid-specific genes TTF1 and PAX8 in TPC1 cell line, but not in other cell lines (e.g., BHP 10-3, and ARO) (70). TTF-1 and PAX8 are thyroid-specific transcription factors and are crucial for thyroid development and function. These transcription factors have been known to regulate the expression of various thyroid specific genes which include Tg, NIS thyroid peroxidase (TPO), and thyrotropin receptor (TSHR) (71). The low levels of TTF-1 and loss of its nuclear localization in thyroid cancer have been associated with dedifferentiation and increased malignancy, whereas the role of PAX8 in thyroid cancer is still controversial (71, 72). The lithium-mediated induction in the expression of thyroid-specific transcription factors upon Wnt activation, suggest these transcription factors as downstream targets of Wnt/β-catenin pathway in thyroid cancer cells (Figure 1).

Another study also documented the role of lithium in the differentiation of a follicular thyroid cancer (FTC) model. FTC is characterized by a significantly lower expression of Nuclear Receptor Subfamily 4 (NR4A1) compared with benign follicular adenomas (73). NR4A1 receptors have emerged as important molecular switches in processes associated with carcinogenesis, including apoptosis, DNA repair, proliferation, migration, inflammation, and metabolism. NR4A1 has been shown to be lower in various tumor types in comparison to normal tissues. The Wnt signaling pathway is one of the upstream mechanism of NR4A1 regulation (74). Lithium treatment resulted in restoration of NR4A1 expression in FTC cell line as well as inhibition of cell growth and induction of apoptosis *in vitro* (73). Besides NR4A1, lithium treatment upregulated the expression of FOSB which is a downstream target of Wnt signaling pathway. This suggests that upregulation of NR4A1 by lithium treatment is possibly associated with activation of Wnt signaling pathway. Wnt signaling pathway is known to be regulated by GSK-3β (75) and lithium treatment has been shown to inhibit activity of GSK-3β (76). The exact mechanisms by which lithium inhibits GSK-3β and Wnt regulates NR4A1 are not known. Moreover, one of the cell lines used in this above mentioned study was WRO, and its classification as a model for human FTC has been questioned in subsequent studies (77).

Since lithium is a potential differentiation stimulus, several studies have tested its role as an RAI uptake enhancer in *in vitro* models via induction of NIS. The main shortcoming of this approach is that cancer cells lack the machinery responsible for iodine organification and therefore intracellular RAI is rapidly released (78). Liu et al. analyzed the role of lithium in a rat follicular cell line intrinsically expressing NIS (FRTL5), and a follicular thyroid cancer cell line FTC133 stably transfected with NIS (60). Lithium in a concentration ranging between 0.5 and 2 mM did not affect RAI efflux nor residence time in these models (60). Similarly, Elisei et al. used anaplastic and medullary thyroid cancer models stably transfected with NIS and showed that treatment with 10 mM of lithium chloride was not associated with increased RAI retention (78).

More encouraging results have been found in both *in vitro* and *in vivo* models of medullary thyroid cancer (MTC). Lithium at concentrations ranging between 10 and 30 mM, through inhibition of GSK-3β signaling, led to significant inhibition of MTC cell line growth and decreased production of neuroendocrine markers (79). The growth inhibitory effects of lithium were associated with increase in the levels of cyclin-dependent kinase inhibitors (p21, p27, and p15) leading to cell cycle arrest. The lithium mediated growth inhibition was aslo confirmed in an *in vivo* xenograft model of MTC (79). Moreover, these effects were achieved with therapeutic concentrations in mice sera, ranging between 0.2 and 1 mEq/l, thus reflecting the therapeutic window in humans. Adler et al. documented an additive effect of treatment with lithium in combination with the histone deacetylase inhibitor valproic acid in inhibition of growth and induction of apoptosis of TT (MTC cell line) cells *in vitro* (14).

Overall, based on *in vitro* experiments, there is evidence that lithium affects GSK-3β and Wnt/β-catenin signaling in a cell type-dependent manner—with stimulation of proliferation of normal follicular cells and inconsistent inhibition of cell growth in thyroid cancer cell lines (Figure 1; Table 1). The majority of pre-clinical studies utilized *in vitro* models exposed to supra-therapeutic concentrations of lithium, limiting its translational perspective. The only *in vivo* data documenting growth inhibitory effects of lithium are available for an MTC xenograft model. The role of lithium as a differentiation stimulus and RAI-retention enhancer is limited and needs to be clarified based on *in vivo* studies.

**Table 1:** Effects of lithium on different endocrine tumors as stated in pre-clinical studies.

**Endocrine tumors**	**References**	**Preclinical model**	**Lithium dose**	**Study endpoint**
Papillary thyroid cancer	(20)	TPC1 cell line, human PTC tumors	5 mM, 20 mM	Induction of TTF1 expression
Follicular adenoma and carcinoma	(66)	S18, NPA, FTC133	5–20 mM	Stimulation of proliferation via inhibition of GSK-3β and activation of Wnt/beta-catenin signaling
Follicular and anaplastic thyroid cancer	(73)	WRO, NPA, ARO	10–20 mM	Restoration of *NR4A1* expression, inhibition of growth, induction of apoptosis
Papillary and anaplastic thyroid cancer	(70)	TPC1, BHP10-3 ARO,	20 mM	Differential cell type-based response to lithium TPC1—upregulation of TTF1 and PAX8 BHP10-3, ARO—downregulation of TTF1 and PAX8
Follicular thyroid cells and follicular thyroid cancer	(60)	FRTL5, FTC133	0.5–2 mM	Failure to induce enhanced RAI retention
Medullary and anaplastic thyroid cancer	(78)	TT, FRO stable transfected with NIS	10 mM	Failure to induce enhanced RAI retention
Medullary thyroid cancer	(79)	TT cell line, TT xenograft	*in vitro*-−5–30 mM, *in vivo*-−340 mg/kg body weight	Inhibition of growth via inhibition of GSK-3β
Medullary thyroid cancer	(14)	TT cell line	15–20 mM	Inhibition of growth, additive effect of HDAC inhibitor, induction of apoptosis
Parathyroid adenoma	(102, 103)	HEK293 and/or parathyroid adenoma derived cell line	Variable	Activation of Wnt/beta-catenin signaling pathway leading to an inhibition of parathyroid embryonic transcription factor TBX1
Parathyroid adenoma	(104)	Primary cultures of parathyroid adenomas	2 mM	Lithium -induced increased proliferation of parathyroid cells
Parathyroid hyperplasia and adenoma	(105)	Normal and hyperplastic parathyroid glands, parathyroid adenomas	1.3 mM	Lithium induced PTH excretion in normal and hyperplastic parathyroid tissue
Adrenal Cortex Tumor	(110)	Adrenal cortex tumor and normal human tissues	5, 10, and 50 mM	Inhibition of DNA Fragmentation
Pituitary Tumor	(123, 124)	AtT-20 cell line	Variable	Pre-treatment with lithium inhibited ACTH secretion upon subsequent lithium exposure
Neuroendocrine tumor	(119)	BON and NCI-H727 cell line	20 mM	Inhibition of cellular growth and inactivation of GSK-3β
Neuroendocrine tumor	(120)	BON and NCI-H727 cell line	0–50 mM	Dose-dependent reduction in cancer cell proliferation, induction in apoptosis and inactivation of GSK-3β
Pheochromocytoma	(111)	PC12 cell line	0.5 mM	Promotes cell growth and protects cells from toxic compounds like thapsigargin and trimethyltin
Pheochromocytoma	(16, 112)	PC12 cell line	Variable	Dose-dependent cytotoxic effects
Pheochromocytoma	(113–115)	PC12 cell line	Variable	Pretreatment with lithium protects against morphine, beta-amyloid peptide and hydrogen peroxide-induced cell death

### Clinical Evidence

#### Lithium and Benign Thyroid Nodules/Goiter

Turner et al. documented increased thyroidal retention of RAI in patients with hyperthyroidism treated with lithium (54) (Table 2). Data from two large randomized studies performed in Italy revealed that lithium enhanced the effectiveness of RAI therapy resulting in a more prompt control of the disease in patients with large goiters (55, 56). The authors also claimed that lithium diminished RAI-induced release of thyroid hormones into the circulation, an observation confirmed by another group (58), suggesting that adjuvant lithium therapy could be employed in the RAI therapy for goiters, particularly in elderly patients with high cardiovascular risk. Martin et al. also showed that cure was twice as likely in patients with toxic multinodular goiter receiving RAI with adjuvant lithium compared with RAI therapy alone (80). However, another randomized study concluded that the role of lithium as adjuvant therapy in patients with large goiters was insignificant (57). The latter study was the largest one involving patients with goiters (Table 2), suggesting that the role of lithium as an adjunct to RAI therapy might be either minimal or not significant. The discrepancy in the results of above-mentioned studies might be due to varying RAI dosages implemented in therapy, different thyroid volumes of included patients, varying doses, and duration of exposure to lithium, different iodine nutritional status, to name just a few. Interestingly, exposure to lithium has been associated with morphological changes of the thyrocytes, namely pronounced pleomorphism of the epithelial cells and marked nuclear changes (81). These features might be interpreted in cytology specimens received from fine-needle aspiration biopsy of thyroid nodules as atypia of uncertain significance (82). Therefore, lithium may need to be added to the list of medications causing cytologic atypia.

**Table 2:** Clinical studies utilizing lithium in benign thyroid disorders and malignant thyroid nodules.

**Endocrine tumors**	**References**	**Study design**	**Lithium dose**	**Study endpoint**
**BENIGN THYROID DISEASE**				
Goiter	(54)	Prospective cohort study−16 patients RAI + Lithium, 16 patients—RAI alone	400 mg post-operative daily 7 days before and 7 days after RAI	Prolonged retention time of RAI in lithium-treated group
Goiter due to Graves disease	(55)	Randomized controlled prospective study 55 patients—RAI 55 patients—RAI + Lithium	900 mg daily for 6 days after RAI	Earlier cure from hyperthyroidism, no difference in treatment efficacy between RAI alone and RAI + Lithium at the end of the study
Goiter due to Graves disease	(56)	Prospective cohort study−36 patients with Graves disease *N* = 12—RAI alone *N* = 12 Lithium for 6 days *N* = 12 Lithium for 19 days	Lithium 900 mg/daily for 6 days post-RAI (*n* = 12) Lithium 900 mg/daily for 19 days post-RAI (*n* = 12)	Patients treated with RAI plus lithium had a prompter control of hyperthyroidism than patients treated with RAI alone
Graves disease and multinodular goiter	(57)	Randomized controlled prospective study−152 patients treated with RAI alone 164 with RAI + Lithium	300 mg three times a day, for 3 weeks starting on the day of radioiodine administration	No difference in the success rate in RAI group vs. RAI + Lithium group
Multinodular goiter	(58)	Randomized controlled prospective study−35 patients RAI alone, 33 patients RAI + Lithium	900 mg daily for 6 days post-RAI	Low incidence of RAI-induced thyrotoxicosis in patients from RAI + Lithium group. No difference in a degree of reduction of goiter size between RAI and RAI + Lithium group
Toxic multinodular goiter and Graves disease	(80)	Retrospective cohort study 110 patients RAI alone, 123 patients RAI + Lithium	800 mg daily 3 days before and 7 days after RAI	The likelihood of cure 60% greater in the RAI + lithium group compared with RAI alone
**MALIGNANT THYROID TUMORS**				
Follicular thyroid cancer	(83)	Case report	600 mg loading dose, followed by 900 mg daily during diagnostic dosimetry and post-RAI treatment for 4 days	Increased RAI retention within metastatic lesions, increased bone marrow exposure to RAI
Metastatic differentiated thyroid cancer	(84)	Case series−4 cases	Lack of detailed information	Dosimetry available for 1 out of 4 patients—increased residence time of RAI in the metastatic lesion
Metastatic and non-metastatic differentiated thyroid cancer	(85)	Case series−6 patients with metastatic thyroid cancer, 12 patients without metastases	400–800 mg 1 day before and 7 days after RAI treatment	Increased RAI retention in all metastatic lesions, increased RAI retention in half of normal remnant thyroid tissue
Differentiated thyroid cancer	(59)	Prospective cohort study−9 patients with PTC 6 patients with FTC	600 mg loading dose followed by 900 mg post-operative daily to target plasma concentration of 0.6–1.2 mEq/l	Lithium increased 131-I retention in 24 of 31 metastatic lesions and in 6 of 7 thyroid remnants. Lithium prolonged the effective half-life in metastases by more than 50% and increased the estimated RAI dose in the metastatic tumor by 2.29 times
Differentiated thyroid cancer	(87)	Cohort study−41 patients prepared for RAI with endogenous TSH stimulation + lithium, 52 patients—endogenous TSH stimulation without lithium, 42 patients recombinant human TSH stimulation	600 mg loading dose followed by 900 mg daily adjusted to lithium level of 0.6–1.2 mEq/l, given 7 days before and 2 days after RAI	No difference in progression-free survival between the groups, lithium group characterized by the longest overall survival in an unadjusted model, but adjustment by age and disease burden revealed no association between lithium and overall survival
RAI-non responsive metastatic thyroid cancer	(88)	Cohort study−24 patients treated with RAI + Lithium and 48 patients—RAI alone	300 mg daily for 7 days	Improved overall survival in RAI + Lithium group compared with RAI alone
Low risk differentiated thyroid cancer	(89)	Randomized controlled study−32 patients RAI alone, 29 patients RAI + lithium	900 mg daily for 7 days	Higher rate of thyroid remnant ablation at 12 months in RAI + Lithium group compared with RAI alone
Differentiated thyroid cancer	(86)	Randomized double-blinded prospective study of 21 patients TRH aided RAI−7 patients Lithium -aided RAI−6 patients TRH + lithium—aided RAI−8 patients	600 mg daily for 7 days	RAI uptake in the remnant thyroid comparable in all groups
Metastatic and non-metastatic differentiated thyroid cancer	(90)	Cohort study−201 patientsLow-risk rhTSH-aided RAI 30 mCi−44 patients Low-risk rhTSH + Furosemide RAI 30 mCi−45 patients Low risk rhTSH + furosemide + lithium RAI 30 mCi−45 patients High risk rhTSH-aided RAI 100 mCi−20 patients High risk rhTSH + furosemide RAI 100 mCi−22 patients High risk RhTSH + furosemide + lithium RAI 100 mCi−20 patients	450 mg daily for 3 days post-RAI	Significantly better remnant ablation rate in low-risk patients treated with lithium compared with patients not treated with lithium No difference in the rate of remnant ablation in high-risk patients, regardless of the method of preparation
**NEUROENDOCRINE TUMORS**				
Neuroendocrine tumor	(121)	Phase II clinical trial—15 patients with low-Grade NETs	300 mg three times a day for 28 days	Lithium was ineffective in reducing tumor volume in the patients

#### Lithium and Thyroid Cancer

One of the first studies utilizing adjunctive lithium therapy in patients with thyroid cancer were case reports of metastatic thyroid cancer associated with hyperthyroidism due to hormonal overproduction by DTC (83, 84). The effects of addition of lithium were quantified with tumor and whole-body dosimetry. Adjunctive lithium therapy resulted in a longer retention time of RAI within the metastatic lesions but also increased exposure of the bone marrow to radiation (83). Moreover, achievement of therapeutic lithium levels of 0.6–1.2 mEq/l was associated with side effects, including nausea and vomiting. Interestingly, Pons et al. showed that although RAI retention was significantly increased in all metastatic lesions in DTC patients with distant metastases, it was not significantly increased in the remnant normal thyroid tissue in ~50% of low-risk patients (85). This observation was further confirmed by Ang et al. who showed a lack of enhanced retention of RAI in thyroid remnant tissue after exposure to lithium (86).

Another case series from the National Institute of Health, United States of America including 15 patients with metastatic DTC found that lithium increased RAI retention in 24 of 31 metastatic lesions and in 6 of 7 thyroid remnants. Lithium prolonged the effective half-life in metastases by more than 50% and increased the estimated RAI dose in the metastatic tumor by 2.29-fold (59). However, the long-term efficacy of adjunctive lithium therapy in this cohort was not available until recently, when our group summarized the National Institute of Health experience with lithium-aided RAI therapy for metastatic DTC (87). There was no difference in progression-free survival in patients treated with lithium-aided RAI compared with patients treated with RAI alone. Although therapy with lithium was associated with improved overall survival in unadjusted models, adjustment by clinically relevant factors affecting overall survival such as age and disease burden revealed no added benefit of lithium. The main factors affecting the outcome were patient age and disease burden, while the method of preparation for RAI therapy was not associated with improved progression-free or overall survival (87). This study was characterized by the largest to date cohort of dosimetry-based lithium-aided therapy of metastatic thyroid cancer and relatively decent duration of follow of a median 5 years. In a smaller cohort, 24 Korean patients with metastatic RAI-refractory DTC underwent combined treatment with RAI and lithium and the treatment efficacy was compared with 48 patients treated solely with RAI. The comparison analysis was performed using propensity score matching (88). The overall survival was significantly better in lithium-aided RAI treated patients, but the analysis was not adjusted by other factors affecting the outcome such as age, number, and location of metastatic foci (88).

In contrast to patients with high-risk metastatic thyroid cancer, addition of lithium to low dose RAI for thyroid remnant ablation in low-risk DTC patients without distant metastases has proven to be beneficial. Yamazaki et al. performed a randomized controlled study in Brazil involving 32 patients treated with 30 mCi of RAI alone and 29 patients treated with RAI with addition of lithium. One year follow up studies revealed a higher thyroid remnant ablation rate in the RAI + lithium group compared with RAI alone, as documented by undetectable stimulated thyroglobulin and negative ^123^I whole-body scan (89). Similar findings were observed in another study conducted in Italy by Barbaro et al. on thyroid ablation in low-risk DTC patients, while this study found no difference in remnant ablation in high-risk patients treated with 100 mCi of RAI, regardless of whether lithium treatment was added (90). It is worthwhile to speculate that the higher efficacy of lithium-aided RAI therapy in low-risk patients might be due to the higher level of NIS expression in low-risk patients, particularly in normal remnant tissue, compared with relatively lower NIS expression in tissues derived from distant metastases of high-risk patients (91). Lithium-induced extension of RAI residence time in metastatic lesions might not be sufficient to deliver tumoricidal RAI dose to the high-risk tumors. It might be interesting to design a study utilizing combination therapy with NIS inducing agents such as MEK or BRAF inhibitors with lithium as an adjunct to RAI therapy of high-risk DTC patients.

Despite very promising pre-clinical data in *in vitro* and *in vivo* MTC models, a clinical trial utilizing lithium monotherapy in MTC was terminated, and 3 out of 5 enrolled patients died of disease (NCT00582712).

Overall, therapy with lithium was well-tolerated. Gastrointestinal disturbances (nausea, vomiting, diarrhea) were reported most commonly in up to 10–20% of patients (85). Although lithium treatment has been reported to increase remnant ablation rate in low-risk patients with DTC, its utility in this setting may be limited as current guidelines recommend against RAI in thyroid cancer confined to the thyroid gland based upon the observed lack of benefit of RAI therapy on mortality and morbidity in low-risk thyroid cancer patients. Additive lithium therapy in high-risk patients has not been proven to improve long-term outcomes in patients with metastatic DTC.

## Lithium and Parathyroid Tumors

### Epidemiology

Hyperparathyroidism due to benign parathyroid adenomas overproducing parathyroid hormone (PTH) is one of the most common endocrine disorders. Treatment with lithium has been associated with 4–6 higher likelihood of hyperparathyroidism compared with its incidence in the general population (92). Most studies have shown that lithium-induced hyperparathyroidism is associated with the presence of a solitary parathyroid adenoma (92–96). However, lithium-induced polyglandular hyperplasia has also been described (95–97).

The molecular signature of lithium-induced parathyroid adenomas reveals that gross chromosomal alterations occur rarely. In most cases, the tumorigenic pathway is independent of multiple endocrine neoplasia gene (MEN1) and genes at 1p34.3 and 1q21-q32, suggesting a unique etiology of development of these tumors (98).

The management of lithium-induced parathyroid adenomas is predominantly surgical, however, calcimimetics have been also successfully used, particularly in the pediatric population (96, 97, 99–101).

### Mechanism of Lithium Action—Pre-clinical Evidence

Calcium sensing receptors (CaSRs) are present in parathyroid glands, renal tubules, and bones. Lithium is known to shift the CaSR set point in parathyroid cells, raising the threshold of serum calcium necessary to inhibit PTH secretion, resulting in an increase in parathyroid cell proliferation and increased PTH synthesis and secretion (94). One of the potential mechanisms of stimulation of parathyroid cell proliferation by lithium might be the activation of the WNT/β-catenin signaling pathway. Verdelli et al. in an *in vitro* model of parathyroid adenoma-derived cells showed that lithium-induced activation of WNT/β-catenin signaling led to downregulation of the transcription factor TBX1, which is expressed in adult parathyroid cells and deregulated in parathyroid tumors (Figure 1) (102). Similar findings of activation of Wnt/β-catenin signaling pathway leading to downregulation of TBX1 were observed by Corbetta et al. in HEK293 cells (103). TBX1 deficiency may potentially contribute to the low proliferative index of parathyroid tumors (102). In fact, Saxe et al. in a study utilizing primary cultures of parathyroid adenomas showed that therapeutic concentrations of lithium of 2 mM lead to enhanced proliferation of parathyroid cells *in vitro* (104). Lithium has been also shown to stimulate release of PTH *in vitro* (105). To summarize, pre-clinical *in vitro* studies consistently show that lithium induces proliferation and PTH secretion in parathyroid cells (Table 1).

## Tumors of the Adrenal Cortex and Medulla

### Epidemiology

Adrenocortical tumors are common in occurrence with most of the tumors being benign and non-functional adrenocortical adenomas (106). A very small proportion of adrenal tumors known as adrenocortical carcinomas (ACC) are malignant in nature and can cause significant morbidity and mortality (107). ACC is a rare malignancy with an incidence of 0.7–2.0 cases/million inhabitants per year (108). Similar to ACC, pheochromocytomas, and paragangliomas which are tumors of adrenal medulla are considered rare tumors affecting around 3 per million people annually (109). Currently, there is no patient-based information available pertaining to the effects of lithium on ACC, pheochromocytomas, and paragangliomas.

### Mechanism of Action—Pre-clinical Evidence

At present, there is only one pre-clinical study where the effects of lithium were analyzed on adrenal cortex tumors. The study reported dose-dependent inhibition of apoptosis associated with a reduction in DNA fragmentation in tumor as well as adjacent normal tissues procured from the patients with adrenal cortex tumors. In contrast to this, lithium treatment caused enhanced DNA fragmentation in the adrenocortical tissue of patients with Cushing disease (110). This study was very preliminary in nature as the conclusions were drawn from the analysis of DNA fragmentation observed in the agarose gels and the study utilized supraphysiological concentrations of lithium (5, 10, and 50 mM).

Up until now the effects of lithium on pheochromocytomas have been studied on a single cell line that is derived from a rat pheochromocytoma (PC12). Lithium treatment in PC12 cells at a concentration equivalent to the levels detected in the serum of lithium-treated patients (0.5 mM) resulted in an increase in cell number (111). This increase in cell number was not associated with an increase in proliferation as no significant difference in DNA synthesis was observed. Rather, lithium protected PC12 cells from thapsigargin and trimethylation-induced cell death. A higher lithium concentration (5 mM) resulted in a reduction of cell number (111). In another study, lithium treatment had a concentration-dependent cytotoxic effect on PC12 cells. These cytotoxic effects were associated with interactions of lithium ions to amyloid-β monomers, resulting in the formation of β -sheet fibrils which induced toxic effects within the cells (112).

A similar growth suppression effect of lithium was observed in a study where PC12 cells were exposed to supraphysiological concentrations of lithium (5, 10, and 30 mM) (16). Growth suppression at these concentrations was associated with inhibition of GSK-3β activity via its phosphorylation in a concentration-dependent manner and reduction in the production of vasoactive hormones. The exact mechanism by which lithium phosphorylates and inactivates GSK3 is, however, not known.

Lithium treatment (1.2 mM) has been shown to have protective effects against morphine-induced cell death in PC12 cells. The protective effects of lithium were associated with decreased expression of a pro-apoptotic gene (Bax) and increased expression of an anti-apoptotic gene (BCL-2), resulting in reduced apoptosis (Figure 1) (113). A similar study reported the cytoprotective effects of lithium against a β-amyloid peptide in PC12 cells pretreated with lithium (2 mM) for 7 days in association with increased BCL-2 protein levels (114). Another study showed that lithium treatment at 1 mM concentration for 5 days protected PC12 cells from hydrogen peroxide-induced cell death by increasing nuclear translocation of nuclear factor E2-related factor 2 (Nrf2) protein (115). Altogether, these studies suggest protective rather than anti-cancer effects of lithium at therapeutic concentrations. The studies where cytotoxic effects were observed in PC12 cells were conducted at very high lithium concentrations and therefore lack translational significance (Table 1).

## Other Endocrine Tumors

### Epidemiology

The other endocrine tumors include neuroendocrine tumors (NETs) and pituitary adenomas (also known as pituitary neuroendocrine tumors, PitNETs). NETs are a heterogeneous group of tumors which have both neuro- and endocrine properties. NETs affect 2–5 people per 100,000 population annually, but its incidence is increasing every year (116). At present, there is very limited information available regarding the effects of lithium on NETs.

PitNETs are the common neoplasms arising from the pituitary gland, with an estimated prevalence of around 17% (117). PitNETs are histologically diverse based on their cell of origin and the type of hormone secreted (118). PitNETs are mostly benign in nature but can lead to substantial morbidity by hypersecretion of pituitary hormones. More than 50% of PitNETS are functional tumors and secrete hormones (117). Currently, there is no patient-based information available pertaining to the effects of lithium on PitNETs.

### Mechanism of Action—Pre-clinical Evidence

In an *in vitro* study performed on NET cell lines- gastrointestinal (BON-1) and pulmonary carcinoid (NCI-H727) cells, lithium treatment at 20 mM concentration inactivated GSK-3β, inhibited cellular proliferation and caused cell cycle arrest in both cell lines. These effects were even more pronounced when lithium treatment (15 mM) was combined with either valproic acid (3 mM) or suberoyl bis-hydroxamic acid (40 μM), both of which activate the Notch signaling pathway (119). In another study utilizing the same cell lines, lithium caused a dose-dependent reduction in cell growth through inactivation of GSK-3β and increased expression of ADP-ribose polymerase, while suppressing serotonin and chromogranin A cellular levels (120). These studies suggest an anti-cancer effect of lithium in NET cells, however, they are very preliminary in nature and the observations need to be validated through *in vivo* models at therapeutic concentrations. At present, there are no *in-vitro* or *in-vivo* studies where the effects of lithium have been investigated in PitNETs.

### Clinical Evidence

There is only one clinical trial to date that has been conducted in United States of America by the National Cancer Institute (NCI) to analyze the effects of lithium on patients with NETs. The trial included 15 patients with low-grade NETs that were given 300 mg lithium orally 3 times daily for 28 days followed by measurement of tumor response. The study reported that lithium was ineffective in demonstrating any objective responses in these patients and the trial was discontinued early (121).

## Summary and Conclusions

Patients chronically treated with lithium need to be screened for hypothyroidism, goiter, and hyperparathyroidism, as prevalence of these endocrine abnormalities is higher in lithium-treated patients than in the general population. The growth inhibitory effects of lithium in non-medullary TC, pheochromocytoma/paraganglioma and carcinoid were achieved with supratherapeutic concentrations of lithium *in vitro*, thus limiting its translational perspective. Although pre-clinical *in vivo* models of growth inhibitory effects of lithium in MTC were promising, the only clinical trial focused on lithium therapy enrolled only five patients and was ended before the endpoints were met, thus limiting any conclusions of lithium therapy efficacy in MTC. Currently available clinical data on the efficacy of lithium in therapy of endocrine tumors in humans is limited and associated with conflicting results. The most robust data were obtained from clinical trials utilizing combination of lithium and RAI therapy in patients with goiters and thyroid cancer. The lithium dosage utilized in these studies were comparable to the doses used for bipolar and depressive disorders, which ranges from 0.4–2.0g per day (122). Even though treatment with lithium has been associated with the extended retention of RAI in RAI-avid tissues, there is no long-term clinical benefit of lithium therapy in thyroid cancer. Future studies may focus on the potential synergistic role of the combination therapy with NIS-inducing agents such as MEK or BRAF inhibitors to enhance RAI uptake and lithium as an agent extending RAI retention time in the management of metastatic thyroid cancer. More pre-clinical *in vivo* data utilizing therapeutic concentrations of lithium in monotherapy and combination therapy is needed before translation of lithium from bench to the bedside for the management endocrine tumors is pursued.

## Author Contributions

JK-G: review concept and design. JK-G, ST, and AT: manuscript preparation. JK-G and ST: manuscript review.

### Conflict of Interest

The authors declare that the research was conducted in the absence of any commercial or financial relationships that could be construed as a potential conflict of interest.
